# Optimized peptide extraction method for analysis of antimicrobial peptide Kn2-7/dKn2-7 stability in human serum by LC–MS

**DOI:** 10.2144/fsoa-2022-0013

**Published:** 2022-07-20

**Authors:** Wen Chen, Dickson Kirui, Nancy J Millenbaugh

**Affiliations:** 1 Craniofacial Health & Restorative Medicine, Naval Medical Research Unit San Antonio, 3650 Chambers Pass, Building 3610, Joint Base San Antonio-Fort Sam Houston, TX 78234, USA

**Keywords:** antimicrobial peptide, liquid chromatography, mass spectrometry, peptide extraction, serum stability

## Abstract

**Aim:**

To develop an extraction protocol and determine stability for antimicrobial peptide (AMP) Kn2-7 and its d-enantiomer dKn2-7 in human serum.

**Materials & methods:**

We compared use of ethanol, acetonitrile, RapiGest SF Surfactant and 1% formic acid in ethanol for AMP recovery from serum prior to liquid chromatography-mass spectrometry quantification.

**Results:**

Precipitation of samples with 1% formic acid in ethanol caused the least amount of AMP loss during extraction from serum. Time-course experiments revealed dKn2-7 was significantly more stable than Kn2-7 in 25% serum, with 78.5% of dKn2-7 and only 1.0% of Kn2-7 remaining after 24 h at 37°C.

**Conclusion:**

The optimized method significantly increased peptide recovery and allowed more accurate and consistent quantification of Kn2-7 and dKn2-7 serum stability.

## Plain language summary

Antimicrobial peptides are a new class of molecules being studied for treatment of infections. These peptides can easily be broken down by enzymes present in the body. Removal of the peptides by the enzymes might limit the effect of the drugs against an infection. Our work discusses the importance of testing the stability of these peptides in human serum, a bodily fluid that contains a large amount of enzymes. We describe a method to decrease loss of two potential peptide drugs during sample processing. Further, we report results of testing the stability of these two peptide drugs in human serum.

## Tweetable abstract

Peptide extraction was optimized for maximum recovery of antimicrobial peptides Kn2-7/dKn2-7 from serum for LC–MS analysis. Time course experiments revealed d-amino acid analogue antimicrobial peptides were more stable against host proteases.

Worldwide overuse of antibiotics has led to multidrug resistance in bacteria [1]. Serious infections caused by antibiotic resistant bacteria pose a tremendous threat to public health [2] and create an ongoing demand for development of new antimicrobial agents [3]. Antimicrobial peptides (AMPs) have recently been identified as promising targets for novel drug development, mainly due to their function in the innate immune response, low toxicity and widely demonstrated activity against multidrug resistant bacteria, viruses and fungi [4–6]. One such AMP, Kn2-7, is a synthetic derivative of the wild-type scorpion venom peptide BmKn2 with improved *in vitro* efficacy against Gram-positive and -negative bacteria, including antibiotic resistant clinical isolates [7]. Kn2-7 also exhibited antibacterial activity and enhanced wound healing in a murine skin infection model [7]. Current studies in our laboratory are focused on investigating the antifungal properties of Kn2-7 and its d-amino acid analogue, dKn2-7, as candidates for incorporation into topical formulations for improved treatment of wound infections. This d-isomer is being tested as a strategy for improving stability of the peptide against proteolytic inactivation. Our recent *in vitro* studies have shown dKn2-7 has greater antifungal potency against *Candida albicans* biofilms than Kn2-7 [8]. One possible explanation for the increased antifungal efficacy of dKn2-7 is that d-amino acid peptides are less prone to degradation by endogenous fungal proteases than their corresponding L forms. These data indicate that dKn2-7 may also be more stable against host proteases, such as those present in serum and wound exudate.

Peptide stability in human serum can be studied using liquid chromatography (LC) and mass spectroscopy (MS) techniques. However, the existence of high-abundance proteins and the large dynamic range of protein concentrations make any quantitative analysis of peptides in serum challenging [9]. Furthermore, analyses of peptides are difficult because of carrier protein binding and other nonspecific binding (NSB). The amphiphilic properties of AMPs like Kn2-7 and dKn2-7, which are required for attachment to and insertion into the cytoplasmic membrane of target cells, increases the likelihood of NSB. The development of a successful LC–MS assay method, therefore, is extremely difficult as it requires scrutiny and optimization of the whole workflow for each peptide. In order to investigate d-amino acid modifications to AMPs that could increase their stability against proteases, and hence their duration of activity in a wound environment, an optimized peptide extraction protocol was developed for LC–MS analysis of Kn2-7 and dKn2-7. This improved protocol was then used to evaluate of the stability of these AMPs in the presence of human serum (wound fluid mimic).

## Materials & methods

### Materials

Internal standard AMP 1018 (VRLIVAVRIWRR) was purchased from Genemed Synthesis (TX, USA). Kn2-7 (FIKRIARLLRKIF) and its d-type amino acid isomer dKn2-7 were purchased from GenScript (NJ, USA). Kn2-7 and dKn2-7 stock solutions were prepared in deionized water at 100,000 μg/ml and then stored at -80°C. Kn2-7 and dKn2-7 working solutions were prepared by dilution in deionized water to 1000 μg/ml. Acetonitrile (ACN), methanol and formic acid (all from Sigma-Aldrich, MO, USA) were of MS grade. Human serum and Roswell Park Memorial Institute (RPMI) culture medium containing 165 mM MOPS buffer (pH 7.0) were purchased from Sigma-Aldrich, and RapiGest SF Surfactant was purchased from Waters (MA, USA). High performance LC grade water was used throughout the study.

### Sample preparation

Pilot experiments were first conducted to optimize recovery of peptides from samples. For these pilot experiments, dKn2-7 (final concentration of 50 μg/ml) was mixed with 25% human serum in RPMI culture media. One set of pilot samples was used to determine if incubation of the dKn2-7/serum mixture with a mass spectrometry-compatible surfactant, RapiGest SF surfactant, prior to processing helps reduce losses due to binding of peptides to serum proteins or other factors during precipitation. We also compared ethanol and ACN as precipitants in these experiments. The samples were either incubated with RapiGest SF Surfactant [10] or vehicle (water), and then serum proteins were removed by precipitation with ethanol or ACN. In detail, samples treated with RapiGest SF were prepared as RPMI with serum (950 μl) + dKn2-7 (50 μl) + 100 μl RapiGest SF; control samples without RapiGest SF treatment were prepared as RPMI with serum (950 μl) + dKn2-7 (50 μl) + 100 μl water. Samples were vortexed to mix and incubated at 50°C for 30 min. A 200 μl aliquot of each sample was mixed with 400 μl of ethanol or ACN for peptide extraction. Samples were incubated on ice for 15 min and then centrifuged at 18,000 g for 10 min. A 150 μl aliquot of the supernatant was recovered and mixed with 3 μl of formic acid. According to the RapiGest SF user guide, samples were vortexed to mix, incubated at room temperature for 45 min for complete RapiGest SF degradation [11], and then stored at -20°C.

A separate set of pilot samples (no RapiGest SF treatment) was used to compare the amount of dKn2-7 loss for two different solvents utilized for serum protein precipitation prior to liquid chromatography-mass spectrometry (LC–MS) analysis, in other words, ethanol [12] or 1% formic acid in ethanol [13,14]. Samples were prepared as RPMI with serum (950 μl) + dKn2-7 (50 μl); positive controls were prepared as water (950 μl) + dKn2-7 (50 μl). A 200 μl aliquot of each sample was mixed with 394 μl of ethanol + 6 μl of formic acid or 400 μl of ethanol alone for peptide extraction. Samples were incubated on ice for 15 min and then centrifuged at 18,000 g for 10 min. Supernatants were transferred to a fresh 500 μl tube and stored at -20°C.

After parameters were optimized to reduce peptide loss during sample processing, the extent of AMP degradation by proteases in human serum was determined via a time-course experiment. Kn2-7 or dKn2-7 (50 μg/ml final concentration) was mixed with RPMI media alone or RPMI containing 25% human serum and incubated at 37°C in a water bath for up to 24 h. At predetermined time points (t = 0, 0.5, 1, 2, 3, 4 and 24 h), a 200 μl aliquot of each sample was collected and mixed with 400 μl of 1% formic acid in ethanol. Samples were incubated on ice for 15 min and centrifuged at 18,000 g for 10 min. Supernatants were transferred to fresh 500 μl tubes and stored at -20°C.

### LC–MS conditions

Samples were diluted eight-times with 3% ACN and 0.1% formic acid in water and spiked with internal standard AMP 1018 [15,16] at 1 μg/ml immediately before LC–MS analysis. A 10 μl aliquot of each sample was analyzed using an Agilent ZORBAX Eclipse XDB 80Å C18 column (2.1 × 50 mm, 5 μm, Agilent, CA, USA) [17] and a Shimadzu LC-20AD system (Shimadzu Corporation, Kyoto, Japan) coupled with a SCIEX API 4000 triple quadrupole mass spectrometer (AB Sciex, MA, USA) [16,18].

A two-component LC system was used and consisted of mobile phase A (0.1% formic acid and 10% ACN in water) and mobile phase B (0.1% formic acid in 100% ACN). The flow rate was set at 0.4 ml/min. The ZORBAX Eclipse XDB 80Å C18 column at 40°C was allowed to equilibrate with 100% mobile phase A. Peptides were loaded to the column and washed for 0.5 min with 100% mobile Phase A. Analytes were separated using a linear gradient of 0–90% mobile Phase B for 2.5 min. Between 3.0 and 4.5 min, the mobile phase was set at 90% B. From 4.51 to 7.0 min, the mobile phase was set at 100% A. Retention times for Kn2-7/dKn2-7 and AMP 1018 were 2.44 and 2.40 min, respectively.

MS was conducted in multiple reaction monitoring [18] and positive electrospray ionization mode. Transitions m/z 385.18/239.1 and 385.18/115.1 were used for internal standard AMP 1018 identification, and m/z 558.71/120.0 and 558.71/103.0 for Kn2-7/dKn2-7 identification. Four concentrations of Kn2-7 or dKn2-7 over the range of 0–2 μg/ml spiked with internal standard AMP 1018 at 1 μg/ml were used to prepare calibration curves. The calibration curves were plotted using weighted (1/x^2^) least-squares linear regression analysis of peptide-to-internal standard peak-area ratios versus concentration. AMPs Kn2-7/dKn2-7 and 1018 were quantified with the most abundant multiple reaction monitoring transitions m/z 558.7/120.2 and 385.18/239.1, respectively. The MS/MS settings were: declustering potential (DP) 75 V, collision energy (CE) 39.0 V and collision cell exit potential (CXP) 14.0 V for m/z 385.2/239.1; DP 75 V, CE 45.0 V and CXP 20.0 V for m/z 385.2/115.1; DP 88 V, CE 65.72 V and CXP14.07 V for m/z 558.7/120.0; DP 88 V, CE 108.1 V and CXP 17.0 V for m/z 558.7/103.0. Source and gas parameters were set as: CAD (collision gas) 10 psi (nitrogen); CUR (curtain gas) 20 psi (nitrogen); GS1 (ion source gas 1) 35 psi (nitrogen); GS2 (ion source gas 2) 30 psi (nitrogen); IS (ionspray voltage) 5000 V; TEM (source temperature) 500°C.

### Data analysis

For graphs showing LC–MS chromatograms, the two highest peaks for each peptide are included for identification, and Y-axis maximum values were matched to the highest peak intensity. The peak area of the highest peak for each peptide was used for quantification. Data for Kn2-7 and dKn2-7 stability in media or media with human serum over time are from three independent experiments (n = 3) and are presented as the mean ± SD. Statistical significance was determined using two-way analysis of variance (ANOVA) and *post hoc* Tukey tests with p ≤ 0.05 considered significant. GraphPad Prism software (v. 6.07, GraphPad Software, Inc., CA, USA) was used for statistical analyses.

## Results & discussion

### Peptide recovery

Pilot experiments were conducted to optimize recovery of peptides from human serum because initial testing using 67% ethanol for protein precipitation as described in a previously published method [19] indicated variable losses of up to approximately 50 and 85% for Kn2-7 and dKn2-7, respectively, during sample processing. We hypothesized that peptide losses during extraction are due mostly to binding to highly abundant serum proteins. Previous reports indicate it is not uncommon for peptides to bind non-specifically to the sample matrix or solid surfaces of glass and plastic containers causing significant reductions in sample recovery during extraction [20,21]. Such NSB can occur at any matrix surfaces for which the peptide, protein, or other large molecule has chemical affinity [22–24]. The main molecular forces supporting NSB are pure ionic interactions occurring between two oppositely charged ions, ion-dipole forces resulting from electrostatic attractions between an ion and a neutral molecule that has a dipole, and attractions between molecules of opposite polarity. NSB causing peptide loss can be reduced by weakening these ionic and polar affinities between the peptide and the matrix molecules, for example, by changing the solution pH.

**Figure 1.**
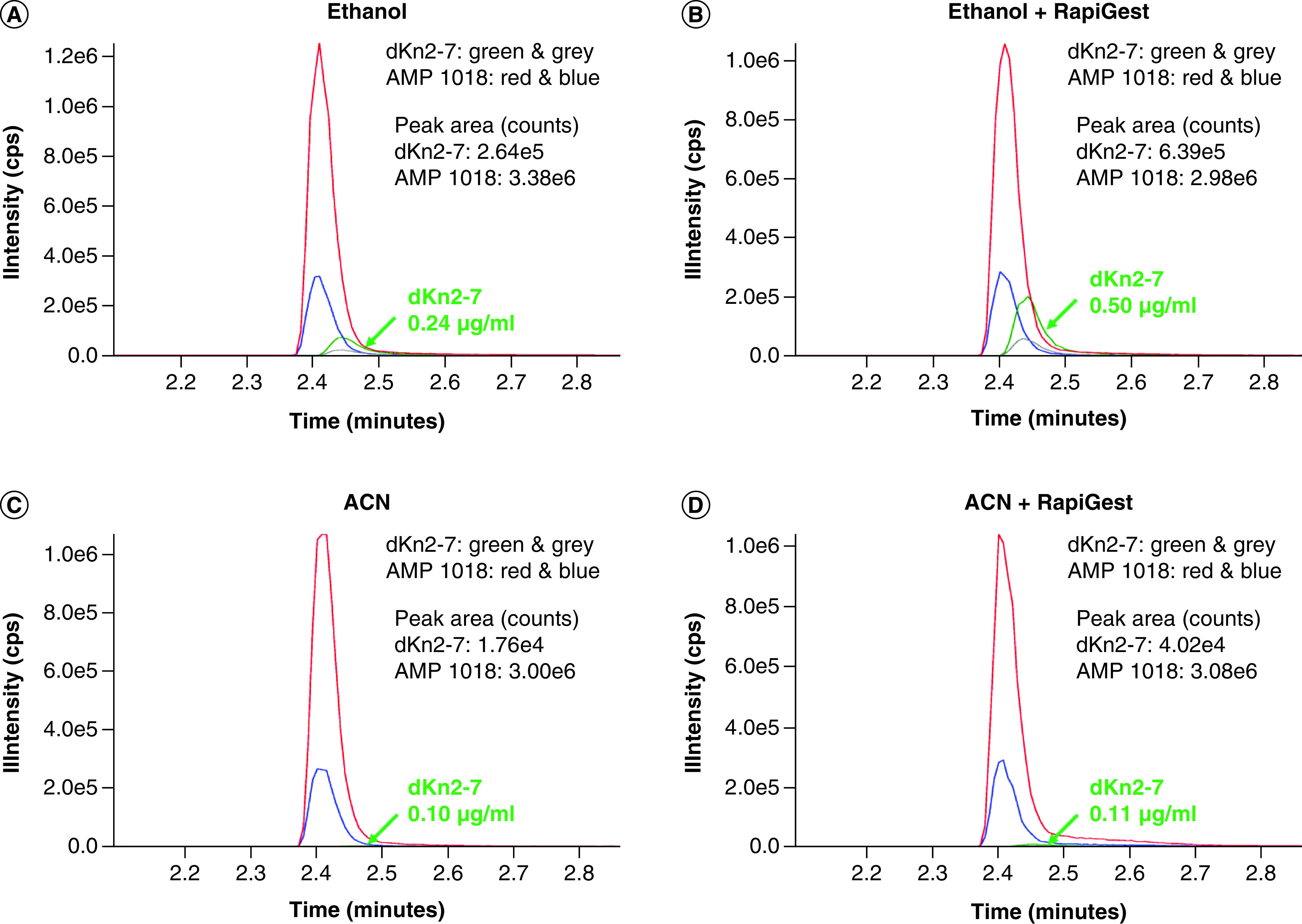
LC–MS chromatograms for dKn2-7 and internal standard AMP 1018. dKn2-7 extraction (green and grey peaks) from RPMI media with 25% human serum was pilot tested using **(A)** ethanol, **(B)** ethanol + RapiGest SF Surfactant, **(C)** ACN or **(D)** ACN + RapiGest SF Surfactant. Peaks for internal control AMP 1018 are in red and blue. The two highest peaks for each peptide are included for identification, and Y-axis maximum values are matched to the highest peak intensity in all graphs. AMP: Antimicrobial peptide.

**Figure 2.**
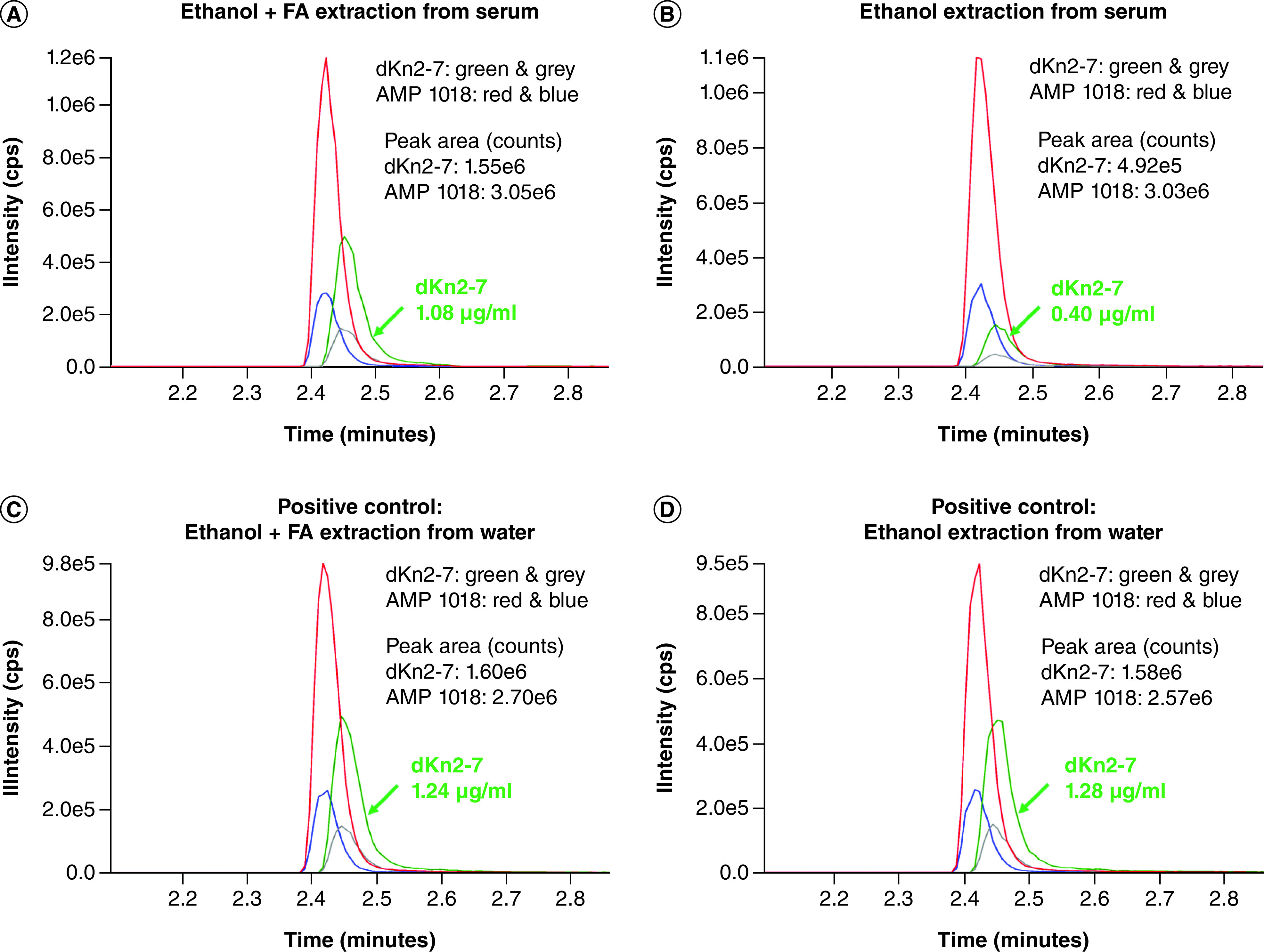
LC–MS chromatograms for dKn2-7 and internal standard AMP 1018. dKn2-7 extraction (green and grey peaks) from RPMI media with 25% human serum using **(A)** ethanol + 1% formic acid (FA) or **(B)** ethanol. dKn2-7 extracted from DI water with **(C)** ethanol + 1% formic acid or **(D)** ethanol were used as positive controls. Peaks for internal control AMP 1018 are in red and blue. The two highest peaks for each peptide are included for identification, and Y-axis maximum values are matched to the highest peak intensity in all graphs. AMP: Antimicrobial peptide.

In our pilot experiments, dKn2-7 was mixed with 25% human serum in RPMI culture media. Addition of RapiGest SF Surfactant only modestly improved recovery of dKn2-7 from serum when ethanol was used as the precipitant and did not improve recovery when ACN was used to precipitate peptides. The amount of recovered dKn2-7 doubled from 0.24 μg/mL for ethanol alone (Figure 1A) to 0.50 μg/ml for ethanol + RapiGest SF Surfactant (Figure 1B); the amount of dKn2-7 recovered was 0.10 μg/ml for ACN alone (Figure 1C) and 0.11 μg/ml for ACN + RapiGest SF Surfactant (Figure 1D). However, precipitation of samples with 1% formic acid in ethanol resulted in a notable increase in the amount of AMP recovered compared with precipitation with ethanol alone, in other words, 1.08 μg/ml (Figure 2A) versus 0.40 μg/ml (Figure 2B) of dKn2-7 recovered from 25% serum, respectively. Also, the amount of dKn2-7 recovered using 1% formic acid in ethanol (1.08 μg/ml) compared well with the amounts recovered from DI water as positive controls, in other words, 1.24 μg/ml (Figure 2C) and 1.28 μg/ml (Figure 2D) for 1% formic acid in ethanol and ethanol alone, respectively. This result can be explained by the fact that the isoelectric point (pI) of most proteins is in the pH range of 4–6. When 1% formic acid is added to a serum solution, proteins are precipitated because, on the acidic side of the pI, they dissociate as cations that combine with anions of formic acid to form salts of the proteins. Our results indicate that the formic acid helped release the AMPs from serum proteins or matrix binding and facilitated protein precipitation. Resultantly, 1% formic acid in ethanol was selected for use in subsequent experiments to evaluate the stability of dKn2-7 versus Kn2-7 in human serum.

### Peptide stability

**Figure 3.**
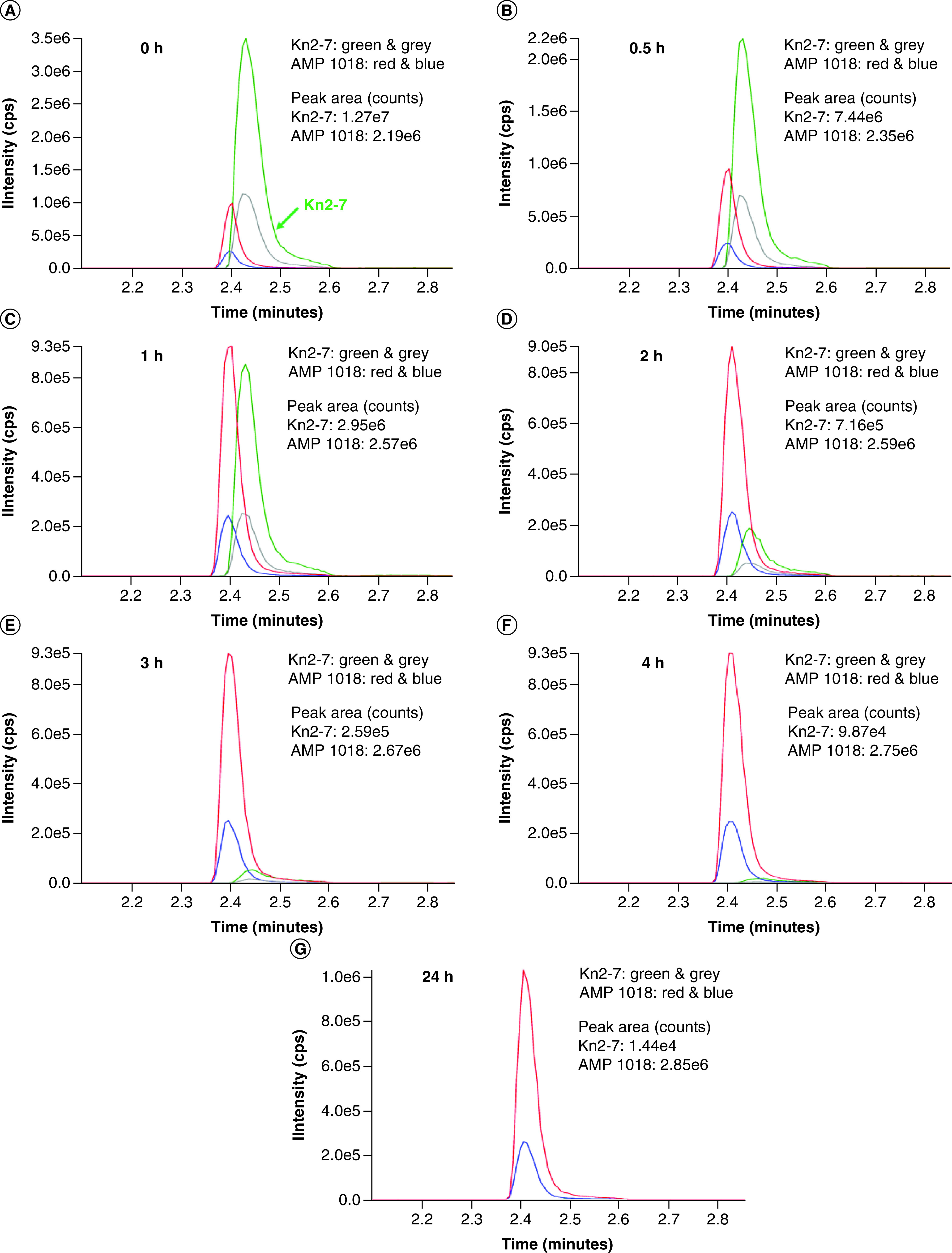
Time-course assay for Kn2-7 stability in 25% human serum. **(A)** Kn2-7 was quantified after 0 h, **(B)** 0.5 h, **(C)** 1 h, **(D)** 2 h, **(E)** 3h, **(F)** 4 h and **(G)** 24 h incubation at 37°C in RPMI media with 25% human serum. Peaks for Kn2-7 are in green and grey, and peaks for internal control AMP 1018 are in red and blue. The two highest peaks for each peptide are included for identification, and Y-axis maximum values are matched to the highest peak intensity in all graphs. AMP: Antimicrobial peptide.

**Figure 4.**
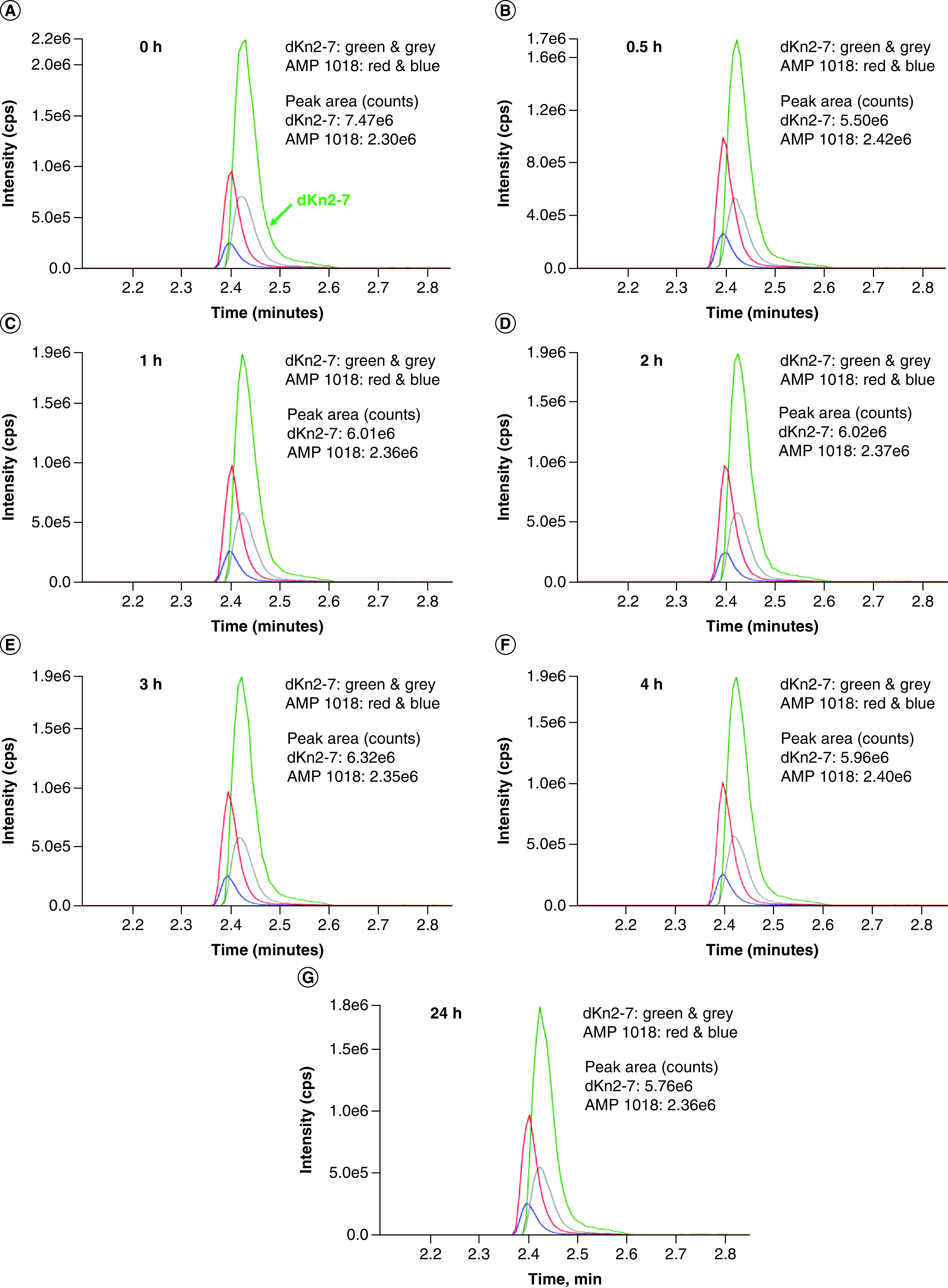
Time-course assay for dKn2-7stability in 25% human serum. **(A)** dKn2-7 was quantified after 0 h, **(B)** 0.5 h, **(C)** 1 h, **(D)** 2 h, **(E)** 3h, **(F)** 4 h and **(G)** 24 h incubation at 37°C in RPMI media and 25% human serum. Peaks for dKn2-7 are in green and grey and peaks for internal control AMP 1018 are in red & blue. The two highest peaks for each peptide are included for identification, and Y-axis maximum values are matched to the highest peak intensity in all graphs. AMP: Antimicrobial peptide.

**Figure 5.**
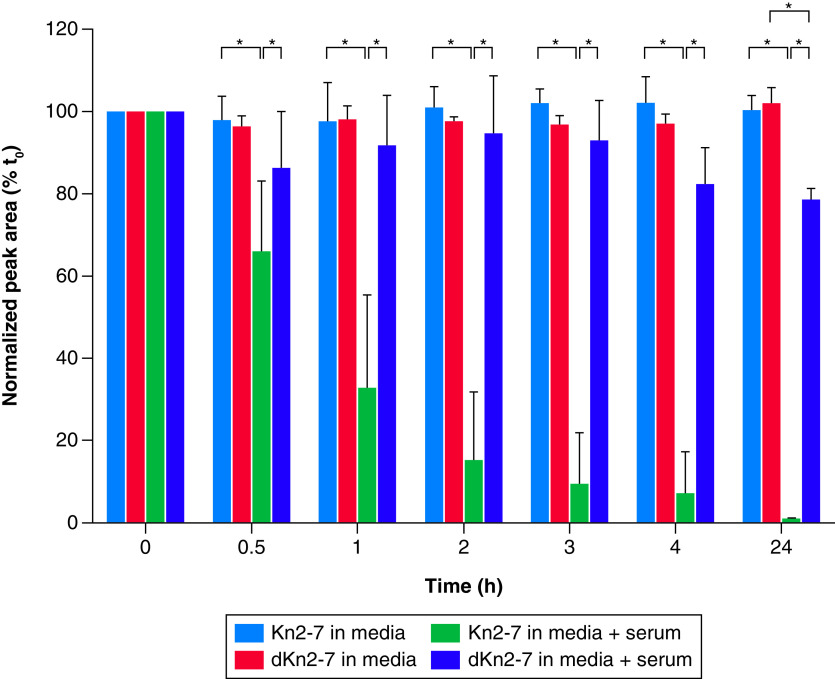
Time-course stability experiments showed dKn2-7 was more stable than Kn2-7 in 25% human serum. After 24 h, 78.5% ± 2.7% (mean ± SD; n = 3) of dKn2-7 remained in the samples, whereas Kn2-7 decreased to only 1.0 ± 0.4% (mean ± SD; n = 3). *p < 0.05 calculated using two-way ANOVA and *post hoc* Tukey tests. ANOVA: Analysis of variance.

The extent of AMP degradation by proteases in human serum was determined by conducting a time-course experiment. Kn2-7 or dKn2-7 at a final concentration of 50 μg/ml was mixed with RPMI media alone or RPMI containing 25% human serum and incubated at 37°C in a water bath for up to 24 h. Results revealed dKn2-7 was significantly more stable than Kn2-7 in human serum (Figures 3, 4 and 5). dKn2-7 concentration decreased to 78.5% ± 2.7%, whereas Kn2-7 decreased to 1.0% ± 0.4% (mean ± SD; n = 3) at 24 h relative to the starting concentration (amount in 0 h sample). These results support our previous hypothesis that the increased antifungal potency of dKn2-7 is related to its higher resistance to proteolytic degradation compared with Kn2-7. Further, the greater stability of dKn2-7 may prolong its activity, and thus increase efficacy against biofilms in a wound environment.

## Conclusion

Quantitative LC–MS analysis of AMPs in biological samples is challenging because of the greater reproducibility and sensitivity needed for accurate determination of low levels of small molecules and complexity of the sample components. AMP loss due to NSB in the sample matrix and at container surfaces is a major problem in quantification due to the highly amphiphilic topologies of this class of peptides [25]. Peptide loss during sample preparation requires mitigation since it often leads to poor reproducibility and less sensitivity in subsequent analysis. In this paper, we consciously optimized various experimental conditions that affect peptide loss. A consistent and efficient protocol for peptide extraction was successfully developed that allowed LC–MS analysis of AMPs Kn2-7/dKn2-7 stability in serum. Furthermore, AMP dKn2-7 was found to be more stable in serum than Kn2-7.

## Future perspective

Resistance to antibiotics is a continual threat to human health with no adequate solutions on the horizon. Every year multidrug resistant microbes infect millions of people worldwide, with many dying of the infection [26]. As promising next generation antibiotics, AMPs are a potential alternative to conventional drugs, are suitable for topical therapeutic applications for treating multidrug resistant infections [27] and pose a lower risk of inducing drug resistance than traditional antimicrobials [28]. Peptides typically do not possess sufficient conformational stability that is essential for use of therapeutic candidates within biological environments [29]; thus, evaluation of peptide stability in serum is a necessary assay for the selection of appropriate leads in the process of AMP drug development. The optimized method described here was developed for Kn2-7 and dKn2-7, which are short peptides with highly basic and amphipathic properties. Further studies are required to confirm our method is applicable for extraction of other AMPs, especially longer peptides and those with fewer positive charges.

**Summary points**

- A reliable and efficient protocol for peptide extraction was successfully developed for LC–MS quantification of antimicrobial peptides Kn2-7/dKn2-7.
- This optimized method significantly increased the peptide recovery and, therefore, allowed more accurate and consistent quantification of the serum stability of antimicrobial peptides Kn2-7 and dKn2-7.
- The stability results support our previous hypothesis that the increased antifungal potency of dKn2-7 is related to its higher resistance to proteolytic degradation compared with Kn2-7.
- Further, the greater stability of dKn2-7 will potentially increase its duration of activity and thus efficacy against biofilms in a wound environment.
